# *Rahnella aquatilis* Sepsis in a Premature Newborn

**DOI:** 10.1155/2015/860671

**Published:** 2015-05-18

**Authors:** Canan Kuzdan, Ahmet Soysal, Hülya Özdemir, Şenay Coşkun, İpek Akman, Hülya Bilgen, Eren Özek, Mustafa Bakır

**Affiliations:** ^1^Istanbul Mehmet Akif Ersoy Thoracic and Cardiovascular Surgery Training and Research Hospital, İstanbul, Turkey; ^2^Marmara University School of Medicine, Department of Pediatric Infectious Diseases, İstanbul, Turkey; ^3^Marmara University School of Medicine, Department of Neonatology, İstanbul, Turkey

## Abstract

*Rahnella aquatilis* is an infrequently isolated Gram-negative rod within the Enterobacteriaceae family. The organism's natural habitat is water. The organism is rarely isolated from clinical specimens and it seldom causes infection in immunocompetent individuals. Here we present a one-month-old boy who was born prematurely at 27th week of gestation by cesarean section with a birth weight of 730 g. He developed sepsis caused by *Rahnella aquatilis* during the treatment for ventilator associated pneumonia due to *Stenotrophomonas maltophilia* with ciprofloxacin. He was successfully treated with a combination of amikacin plus meropenem. Although *R. aquatilis* is one of the *saprophyticus* organisms, it may cause life-threatening infection in newborn.

## 1. Introduction

*Rahnella aquatilis* is an infrequently isolated Gram-negative rod within the Enterobacteriaceae family. The organism's natural habitat is water [1]. The organism is rarely isolated from clinical specimens and it seldom causes infection in immunocompetent individuals. The infections ascribed to this organism are bacteremia, sepsis, respiratory infection, urinary tract infection, wound infections in immunocompromised patients, and infective endocarditis in patients with congenital heart disease. Here we present a one-month-old boy who was born prematurely at 27th week of gestation by cesarean section with a birth weight of 730 g. He developed sepsis caused by* R. aquatilis* despite ciprofloxacin according to ventilator associated pneumonia caused by* Stenotrophomonas maltophilia.* The mother who underwent bone marrow transplantation 3 years before pregnancy for essential thrombocytosis had reportedly preeclampsia during the last trimester.

## 2. Case

He was born prematurely at 27th week of gestation by cesarean section with a birth weight of 730 g, APGAR score 8 at 1 minute and 7 at 5 minutes. He was supported by mechanical ventilator for respiratory distress syndrome and was given parenteral nutrition by an umbilical venous catheter. Because of CRP of 10 mg/L and platelets count of 170,000/*μ*L, ampicillin and gentamicin were initiated for suspected septicemia on the first day of his life. There were not any positive cultures (Table 1). On the second postnatal day, after the increase in both oxygen demand and pressure requirement as well as laboratory findings including leukocytes count of 30,000/*μ*L, platelets count of 80,000/*μ*L, and CRP of 42.4 mg/L, the antibiotic regimen was replaced by vancomycin and cefepime (Table 1). Meanwhile on echocardiography, patent ductus arteriosus was determined; then ibuprofen was added. However ibuprofen was discontinued because of side effects such as increased creatinine and thrombocytopenia.

On the 4th postnatal day, fluconazole prophylaxis was given to prevent invasive fungal infection. On the 8th postnatal day, he was extubated but he needed reintubation on the same day. Despite therapy, we observed deep thrombocytopenia (5,000/*μ*L), leukocytes count of 1,000/*μ*L, CRP of 120 mg/L, and clinical deterioration including abdominal distension and hypotension and, thus, cefepime was replaced by meropenem and liposomal amphotericin B as well as intravenous immunoglobulin. An adjuvant therapy was added to antimicrobial regimen (Table 1). At the same time no pathologic finding was found. Any additional finding with respect to necrotizing enterocolitis was not also found. Then, his clinical picture improved.

However, on the 14th postnatal day, his clinical picture deteriorated again. The umbilical venous catheter was removed. Because he developed suspected pneumonia, the antimicrobial treatment was replaced by linezolid, meropenem, and liposomal amphotericin B. Moreover findings were considered as atelectasis, so the appropriate therapy was given (Table 1). On the 19th postnatal day his clinical picture was noted to improve; therefore he was extubated and any microorganism was not isolated from all cultures, so linezolid was stopped. Meropenem and liposomal amphotericin B were continued.

On the 21st postnatal day apnea and bradycardia occurred. He was supported by mechanical ventilator. Ciprofloxacin was added to liposomal amphotericin B, because* Stenotrophomonas maltophilia* was isolated from endotracheal aspirate culture at the time of clinical deterioration. On the 25th postnatal day, since leukocytes count was 21000/*μ*L, it was determined that platelets count was 33,000/*μ*L and CRP 110 mg/L. Vancomycin was added to antimicrobial regimen, including ciprofloxacin. Liposomal amphotericin B was discontinued. Ceftazidime was added to antimicrobial treatment according to antibiogram susceptibility, because* Stenotrophomonas maltophilia* was isolated in endotracheal aspirate culture again. At the same time echocardiography did not show any pathologic findings suggesting endocarditis and patent ductus arteriosus was closed. Meanwhile* Rahnella aquatilis* resistant to piperacillin and cephalosporins, and susceptible to carbapenems and aminoglycosides, was isolated from both blood cultures taken on 2nd day and 3rd day of ciprofloxacin; the antimicrobial regimen was changed to meropenem and amikacin (Table 1). The patient was successfully treated with a combination of amikacin plus meropenem for 21 days. His clinical picture and laboratory findings returned normal. Upon starting full enteral feeding, he was discharged from the hospital.

## 3. Discussion

*Rahnella aquatilis* is environmental bacteria commonly isolated from water [1]. To our knowledge, there are 18 reports in the literature about human infection caused by* R. aquatilis*. The majority of these suggested that the infection had been accompanied by diabetes mellitus, alcoholism, cancer, AIDS, and immunosuppression secondary to medications, suggesting that the microorganism may have been an opportunistic pathogen. However, 5 reported cases were in nonimmunocompromised patients [1–5]. The organisms were isolated from blood [1–4, 6–12], wounds [5, 8], urine [8, 13–15], the respiratory tract [8, 16], and stool cultures [8]. In our patient, considering the patient's clinical deterioration,* R. aquatilis* was accepted as active agent because it reproduced in the separate blood cultures. The origin of the* R. aquatilis* strain isolated from our patient is unclear. There were no other reported* R. aquatilis* infections in the hospital during his time there. We did not think of an outbreak due to* R. aquatilis,* so newborn intensive care unit water was not tested for* R. aquatilis*. Our patient was successfully treated with a combination of amikacin plus meropenem. Although* R. aquatilis* is one of the saprophyticus organisms, it may cause life-threatening infection in newborn, especially early preterm and very low birthweight babies.

## Figures and Tables

**Table 1:** All the patient's significant laboratory/microbiological findings and the corresponding therapeutical changes.

Postnatal day	Clinical picture	Leukocytes count (/*µ*L)	Platelets count (/*µ*L)	CRP (mg/L)	Culture	Treatment
1st day	Suspected septicemia	40,000	170,000	10	Negative	Ampicillin and gentamicin

2nd day	Increasing in both oxygen demand and pressure requirement	30,000	80,000	42.4	Negative	Vancomycin and cefepime

8th day	Need for reintubation, abdominal distension, and hypotension	1,000	5,000	120	Negative	Meropenem and liposomal amphotericin B as well as intravenous immunoglobulin as adjuvant therapy

14th day	Pneumonia and atelectasis?	23,000	150,000	36	Negative	Linezolid was added

21st day	Pneumonia, oxygen desaturation, increasing ventilation demand, and suctioning requirement	28,000	110,000	89	*S. maltophilia* was isolated from endotracheal aspirate culture	Ciprofloxacin was added to liposomal amphotericin B

25th day	Pneumonia, oxygen desaturation, increasing ventilation demand, and suctioning requirement	21,000	33,000	110		Vancomycin and ceftazidime were added to ciprofloxacin; liposomal amphotericin B was discontinued

26th day					The blood cultures taken on 22nd and 23rd day were resulted in on 26th day. *R. aquatilis* was isolated from blood cultures	Meropenem and amikacin for 21 days
